# Case Report: Tocilizumab as time-limited induction therapy enabling steroid withdrawal and conventional maintenance in refractory cutaneous polyarteritis nodosa

**DOI:** 10.3389/fmed.2026.1861077

**Published:** 2026-05-29

**Authors:** Ka Wei Katty Joo Hu, Fiona Jaederlund, Fernando Lohse, Claudio Karsulovic, Lia Hojman

**Affiliations:** 1 Facultad de Medicina, Universidad del Desarrollo-Clínica Alemana de Santiago, Santiago, Chile; 2 Laboratorio de Inmunomodulación Neuroendocrina, Facultad de Medicina, Instituto de Ciencias Biomédicas, Universidad de Chile, Santiago, Chile; 3 Servicio de Dermatología, Clínica Alemana de Santiago, Universidad del Desarrollo, Santiago, Chile; 4 Servicio de Reumatología, Clínica Alemana de Santiago, Universidad del Desarrollo, Santiago, Chile

**Keywords:** cutaneous polyarteritis nodosa, interleukin-6, mycophenolate mofetil, tocilizumab, vasculitis

## Abstract

**Background:**

Cutaneous polyarteritis nodosa (CPAN) is a chronic, relapsing vasculitis frequently associated with glucocorticoid dependence and the absence of standardized treatment algorithms. Therapeutic decisions are largely empirical and management of refractory disease remains challenging.

**Case presentation:**

We report a patient with refractory CPAN and persistent steroid dependence despite corticosteroids and conventional immunosuppressive therapies. A deliberately planned, time-limited induction strategy with tocilizumab (TCZ) was implemented to achieve rapid disease control and enable steroid withdrawal, followed by maintenance with mycophenolate mofetil (MMF).

**Results:**

TCZ led to rapid clinical remission after two infusions, allowing progressive steroid tapering and discontinuation. After six doses, TCZ was stopped and MMF was introduced as maintenance therapy. The patient remains lesion-free at the time of manuscript submission.

**Conclusion:**

A time-limited IL-6 blockade strategy may allow rapid steroid withdrawal and facilitate transition to conventional maintenance in refractory CPAN. This approach may reduce cumulative exposure to both glucocorticoids and biologic agents.

## Introduction

Polyarteritis nodosa (PAN) is a necrotizing vasculitis affecting medium-sized arteries, with a recognized cutaneous variant, cutaneous polyarteritis nodosa (CPAN), in which disease is primarily limited to the skin. Histopathologically, CPAN is characterized by necrotizing vasculitis involving small- to medium-sized vessels, without granulomatous inflammation and, in primary forms, without significant immune complex deposition.

These features are useful in distinguishing primary CPAN from secondary vasculitic processes and other inflammatory dermatoses. CPAN typically presents with nodules, palpable purpura, livedo reticularis, and ulcerations, most commonly in the lower extremities (1).

Despite its localized nature, CPAN often follows a chronic and relapsing course that may require prolonged immunosuppressive therapy and frequently leads to glucocorticoid (GC) dependence (2, 3). Recurrence rates have been reported in up to 30%−50% of cases despite treatment. However, no standardized treatment algorithm exists, and therapeutic decisions remain challenging in refractory disease (4).

Conventional immunosuppressive agents such as methotrexate (MTX), azathioprine, and mycophenolate mofetil (MMF) are commonly used, but their efficacy is variable and no clear hierarchy has been established (2, 4). In the absence of predictive biomarkers, treatment selection is largely individualized and driven by availability, safety profile, and clinician experience. In patients who develop GC dependence and fail conventional immunosuppressive agents such as MTX, azathioprine, or MMF, management becomes particularly challenging.

At this stage, multiple therapeutic options have been reported, including colchicine, dapsone, and intravenous immunoglobulin, although evidence is limited and responses are variable. Biologic therapies, particularly tumor necrosis factor inhibitors and interleukin-6 (IL-6) blockade, have also been used in refractory cases, supported mainly by small series and extrapolation from systemic vasculitides.

Given the central role of IL-6 in vascular inflammation, IL-6 blockade represents a biologically plausible therapeutic target (5, 6). IL-6 plays a pivotal role in medium-vessel vascular inflammation by promoting endothelial activation, facilitating neutrophil recruitment to vessel walls, and driving effector T-cell differentiation toward pro-inflammatory phenotypes. These interconnected pathways contribute to the perpetuation of vascular injury characteristic of necrotizing arteritis. Tocilizumab (TCZ) has been reported in refractory PAN, although available data remain limited.

Beyond the choice of a specific agent, an additional consideration is whether a time-limited induction strategy using biologic therapy could achieve rapid disease control, enable GC withdrawal, and facilitate subsequent maintenance with conventional immunosuppressive agents. This approach may also allow reconsideration of therapies previously considered ineffective, such as MMF.

Here we describe a deliberately planned, time-limited IL-6 blockade strategy in a patient with refractory CPAN, aimed at achieving rapid steroid withdrawal and facilitating transition to conventional immunosuppressive maintenance, with the goal of minimizing prolonged biologic exposure.

## Case report

**Figure 1:**
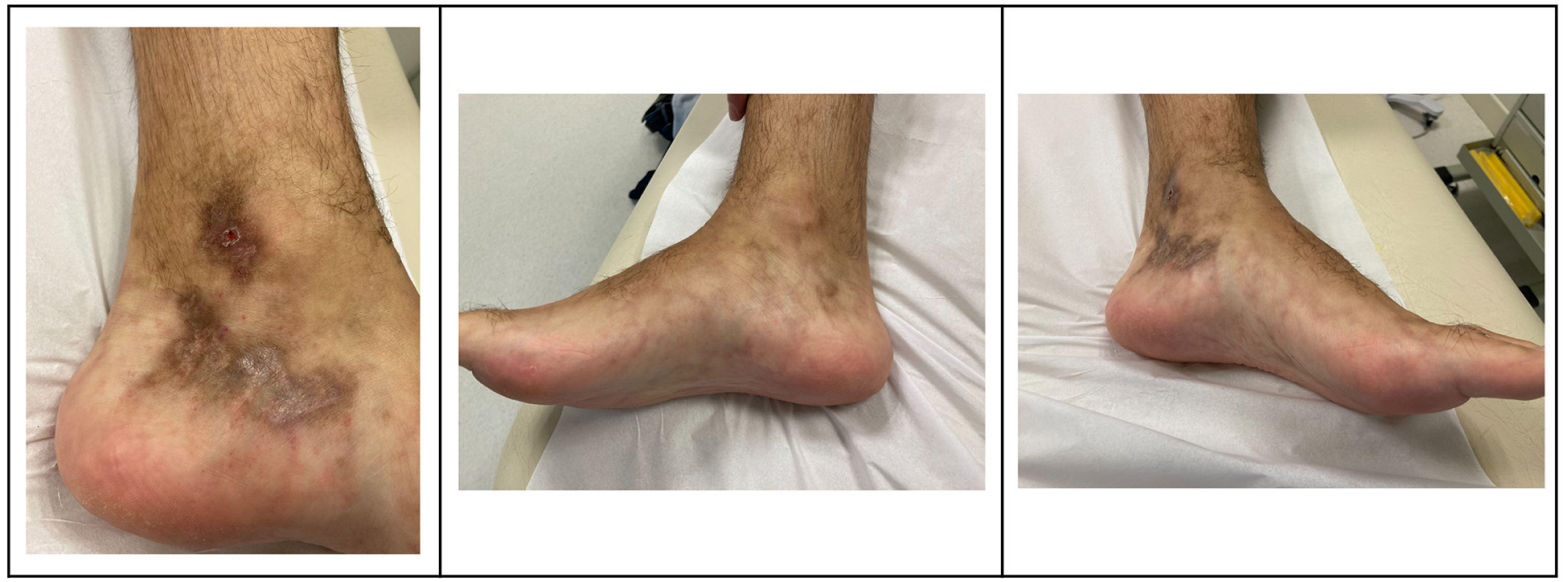
Initial cutaneous lesions on the patient's lower limb. Retiform purpura with an area of ulceration and sclerotic changes of compromised skin on the medial malleolus and more subtle involvement of the lateral malleolus.

A 34-year-old man presented with a long-standing history of recurrent painful skin lesions involving the lower extremities. The patient's medical history was significant for resolved hepatitis B infection, hypertension, and dyslipidemia, managed with amlodipine and atorvastatin, respectively. Serology showed HBsAg negativity and anti-HBc positivity without evidence of active viral replication, making HBV-associated systemic PAN unlikely. The diagnosis of primary CPAN was supported by histopathology, absence of systemic involvement, and normal renal and hepatic function throughout follow-up. Initial lesions were erythematous and purpuric and were misdiagnosed as pigmented purpuric dermatosis. Over time, the disease progressed to livedo racemosa, retiform purpura, and ulceration (Figure 1).

**Figure 2:**
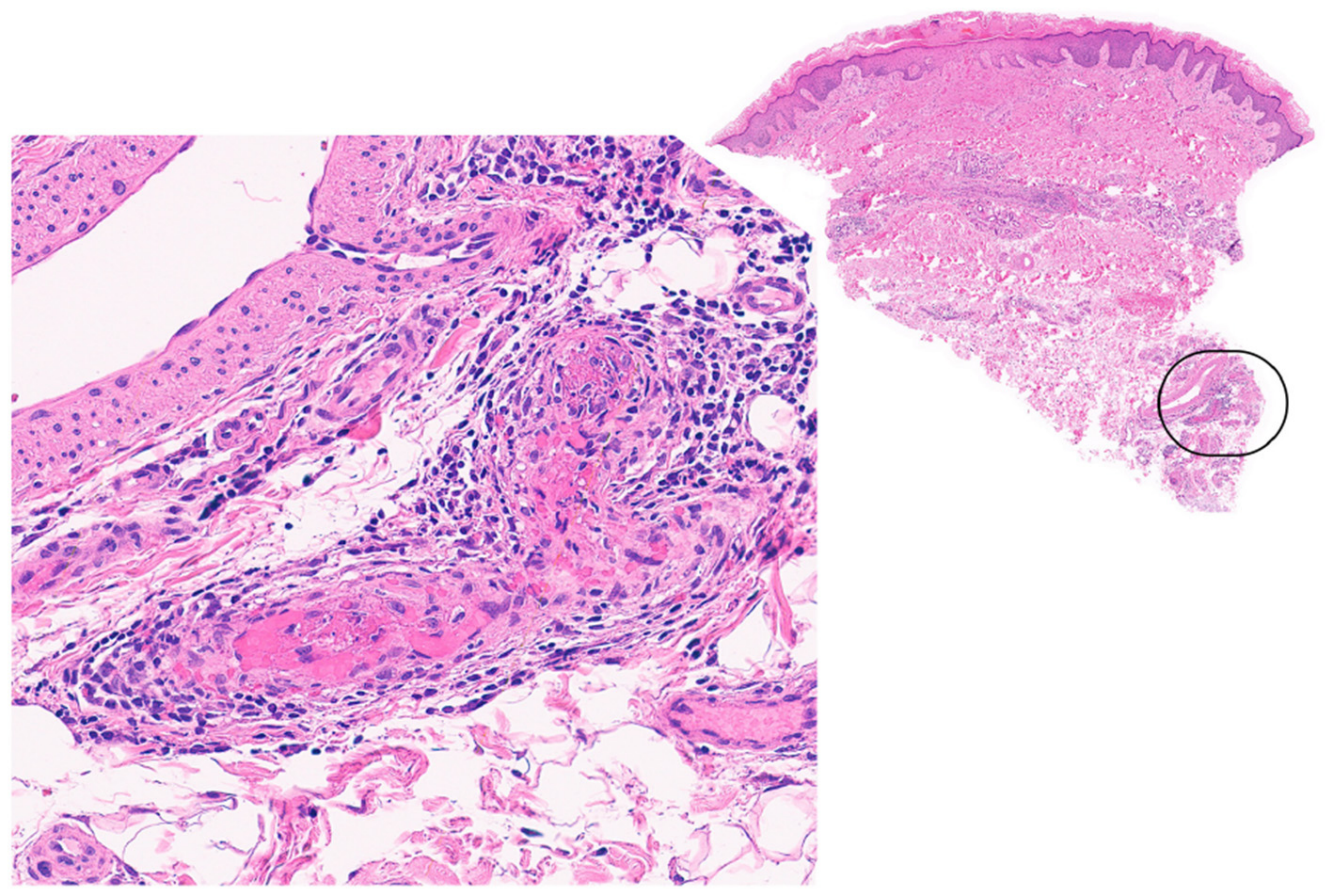
Skin biopsy showing karyorrhexis, thickening of the vessel walls of the superficial plexus, endothelial swelling, fibrinoid degeneration, and microthrombi. In addition, there are numerous venule-capillary vessels and medium-sized vessels with a muscular wall showing fibrinoid necrosis, thrombi, and mural inflammatory infiltrate, consistent with necrotizing vasculitis of small- and medium-caliber vessels.

Extensive evaluation showed no evidence of systemic involvement. Laboratory studies, including inflammatory markers, antineutrophil cytoplasmic antibodies (ANCA), antiphospholipid antibodies, and cryoglobulins, were negative (Table 1). Renal function and urinalysis were normal. Skin biopsy confirmed necrotizing vasculitis of small- and medium-sized vessels, consistent with CPAN (Figure 2). The diagnosis was supported by histopathology, absence of systemic involvement, and longitudinal follow-up. No clinical or imaging evidence of extracutaneous involvement was identified throughout follow-up.

**Table 1:** Results of systemic workup for vasculitis.

Hepatic panel	Creatinine (mg/dl)	CBC	ESR (mm/h)	RF (IU/ml)	ANCA	Anti-B2Gp IgG/IgM	Anticardiolipin Ab IgG/IgM	
*N*	0.86 (NV: 0.7–1.2)	*N*	5 (NV: 2–30)	<10 (NV: <14)	(–)	<3 (NV: <12)	<3 (NV: <12)	
ANA	ENA	Cryoglobulins	Anti-HBc	HBsAg	HCV Ab total	RPR	HIV Ab	IGRA TB
(–)	(–)	(–)	(+)	(–)	(–)	NR	NR	(–)

CBC, complete blood count; ESR, erythrocyte sedimentation rate; RF, rheumatoid factor; ANCA, antineutrophil cytoplasmic antibodies; ANA, antinuclear antibodies; ENA, extractable nuclear antigens; RPR, rapid plasma reagin; IGRA, interferon-gamma release assay; HBsAg, hepatitis B surface antigen; HCV Ab, hepatitis C antibody; Anti-β2GP, anti-beta-2 glycoprotein I antibodies; NV, normal value; NR, non-reactive.

### Therapeutic course

In 2022, CPAN was diagnosed based on clinical presentation and confirmatory histopathology. At diagnosis (December 2022), the patient was initiated on prednisone (PDN) 40 mg/day for 1 month, followed by a planned 6-month taper, in combination with colchicine and pentoxifylline, with an incomplete response. MTX was administered for approximately 1 year, initiated at 15 mg/week and subsequently escalated to 25 mg/week subcutaneously. Despite this, disease control remained suboptimal, and GC dependence was established by mid-2023, with a requirement of approximately 20 mg/day of PDN and multiple unsuccessful taper attempts due to recurrent disease activity.

In 2023, the disease remained active, with persistent livedo racemosa and recurrent ulceration. In addition, the patient developed poor tolerance to MTX, limiting further dose escalation and long-term use. MMF was initiated as a steroid-sparing strategy at a dose of 2 g/day; however, its use prior to TCZ was limited to approximately 38 days due to poor tolerance and lack of clinical improvement in the context of active disease and recent MTX discontinuation. Given ongoing disease activity and persistent GC dependence, a strategic decision was made to avoid further stepwise escalation with conventional immunosuppressive agents and instead pursue a targeted approach aimed at rapid disease control. A time-limited induction strategy with TCZ was therefore implemented to achieve rapid disease control, with the additional goal of minimizing prolonged biologic exposure.

Prior to TCZ initiation, the patient underwent hepatological evaluation. Serology confirmed resolved hepatitis B infection (HBsAg negative, anti-HBc total positive) with no evidence of active viral replication. Prophylactic antiviral therapy was not indicated based on specialist assessment, and no evidence of HBV reactivation was observed during or after TCZ therapy.

In January 2024, TCZ was initiated at a dose of 8 mg/kg intravenously every 4 weeks, at a time when the patient was receiving approximately 30 mg/day of PDN. The patient showed rapid clinical improvement, with complete resolution of active lesions after two infusions. A structured GC taper was implemented during the induction phase, consisting of dose reductions of 5 mg every 2 weeks, allowing complete discontinuation at approximately the time of the third infusion (5–6 weeks). TCZ was administered for a total of 6 monthly doses and then discontinued as planned. Following induction, MMF was reintroduced as maintenance therapy at 2 g/day after a 7-month period without oral immunosuppressive therapy and has been well-tolerated, with no recurrence of prior intolerance. At the time of manuscript submission, the patient remains in sustained remission, without recurrence of lesions.

C-reactive protein (CRP) remained consistently low throughout the disease course (0.06 mg/dl at multiple timepoints), indicating that acute-phase reactants were not informative markers of disease activity in this patient. This observation is consistent with the recognized dissociation between systemic inflammatory markers and cutaneous disease activity in CPAN, and clinical evaluation of cutaneous lesions served as the primary measure of disease activity.

**Figure 3:**
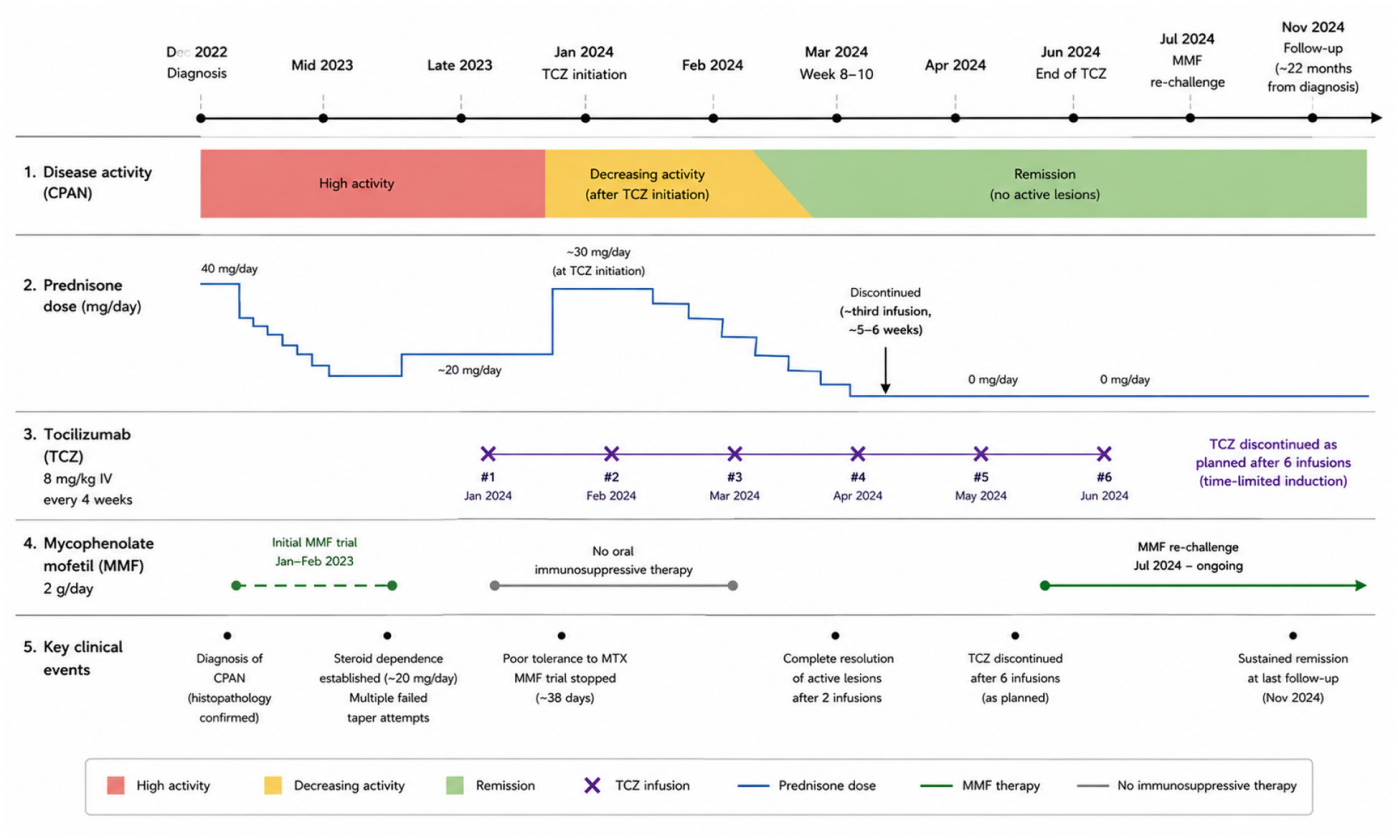
Clinical timeline and therapeutic strategy in refractory cutaneous polyarteritis nodosa. Horizontal timeline illustrating disease activity, prednisone dose, tocilizumab (TCZ) infusions, and mycophenolate mofetil (MMF) exposure from diagnosis through follow-up. High disease activity persisted during 2022–2023 with glucocorticoid dependence. TCZ was initiated in January 2024 as a planned time-limited induction strategy, leading to rapid resolution of active lesions and structured prednisone tapering with complete withdrawal after approximately 5–6 weeks. After 6 monthly infusions, TCZ was discontinued as planned, and MMF was reintroduced as maintenance therapy, with sustained remission at last follow-up (~22 months from diagnosis).

Figure 3 illustrates the clinical timeline, including disease progression, therapeutic interventions, and outcomes.

The rapid clinical response observed after initiation of TCZ should be interpreted in the context of prior therapies. A delayed effect from MTX is unlikely, as it had been used for approximately 1 year without achieving disease control. MMF was administered for a short period (approximately 38 days) prior to TCZ, making a delayed therapeutic effect unlikely. GCs had previously been used at higher doses without sustained response and were being tapered at the time of TCZ initiation. While a contributory effect of prior therapies cannot be completely excluded, the temporal pattern supports a possible role of IL-6 blockade in the observed response.

## Discussion

The rapid clinical response observed after initiation of TCZ must be interpreted with caution. Although a delayed effect of prior therapies cannot be fully excluded, this appears unlikely given the prolonged exposure and lack of efficacy of MTX, the short duration and low dose of MMF, and the absence of sustained response to higher doses of GCs prior to tapering.

Importantly, the decision to initiate TCZ was not solely conceptual but driven by clinical constraints, including persistent disease activity and poor tolerance to high-dose corticosteroids, requiring a rapid steroid-sparing strategy. This distinction is clinically relevant, as it reflects real-world scenarios in which therapeutic decisions are often driven by the need for rapid disease control and treatment tolerability rather than strict adherence to stepwise algorithms.

The use of TCZ in refractory PAN and CPAN has been previously reported in case-based and multicenter evidence, and is supported by current vasculitis guidelines (5–10). Across these reports, TCZ has primarily been employed as rescue therapy or as continuous long-term treatment in refractory disease.

In contrast, the distinguishing feature of our approach lies not in the use of TCZ itself, but in its predefined role within a strategy-driven therapeutic framework. In this case, TCZ was deliberately implemented as a time-limited induction therapy from the outset, with the explicit goals of achieving rapid inflammatory control, enabling GC withdrawal, and facilitating transition to conventional immunosuppressive maintenance, rather than serving as ongoing biologic therapy.

The decision to limit TCZ to six infusions was not based on a fixed protocol but reflected a pre-specified strategic decision to use biologic therapy as a time-limited induction phase rather than as maintenance. Once sustained remission and complete GC withdrawal were achieved, continuation of biologic therapy was not considered necessary. We acknowledge that prolonged biologic therapy is not inherently inferior to prolonged conventional immunosuppression from a safety standpoint; the rationale here was strategy-driven rather than safety-driven, aimed at minimizing cumulative biologic exposure as a secondary goal.

Within this context, the use of a time-limited biologic induction phase followed by conventional maintenance therapy may represent a pragmatic, structured therapeutic approach rather than a drug-specific intervention.

Although CPAN lacks visceral involvement, its chronic and relapsing course often leads to prolonged GC use (2, 3). This is particularly relevant in younger patients, in whom cumulative steroid exposure is associated with significant metabolic, cardiovascular, and skeletal complications.

Current management of CPAN is largely based on low-quality evidence, with no established hierarchy among conventional immunosuppressive agents such as MTX, azathioprine, or MMF (2, 4). Treatment selection is therefore individualized and guided by clinician experience, safety profile, and availability (3). Although MMF had not achieved clinical improvement during its initial short exposure, its success after TCZ-induced remission may reflect several factors, including a delayed therapeutic effect, reduced inflammatory burden, or the natural variability of the disease course. However, this observation should be interpreted with caution, as causality cannot be established in a single uncontrolled case.

The absence of validated treatment algorithms highlights the limitations of a purely stepwise escalation approach and supports the need for more strategy-driven decision making.

IL-6 plays a central role in vascular inflammation, and TCZ has demonstrated efficacy in systemic vasculitides, including PAN (5, 6). In other inflammatory conditions, biologic agents have been used as short-term “bridging” or induction therapies to achieve rapid disease control and facilitate GC tapering. Although evidence for this approach in CPAN is limited, this lack of data highlights an opportunity to further develop strategy-based approaches to disease management. In contrast to prior reports describing long-term biologic use, our case supports the use of TCZ as a time-limited induction strategy, aimed at rapid inflammatory control, steroid withdrawal, and avoidance of prolonged biologic exposure.

This case supports a two-phase, strategy-driven therapeutic model:

Phase 1 (Induction): short-course biologic therapy to achieve rapid suppression of inflammation.

Phase 2 (Maintenance): transition to conventional immunosuppressive therapy for long-term disease control.

This approach may optimize overall therapeutic exposure by reducing reliance on both GCs and prolonged biologic therapy. Importantly, it raises a clinically relevant hypothesis: whether earlier implementation of such an induction strategy could have reduced overall steroid burden in this patient. While this cannot be determined from a single case, it provides a basis for future investigation.

## Conclusion

This case illustrates a strategy-based approach using time-limited IL-6 blockade as induction therapy in refractory CPAN, enabling rapid GC withdrawal and transition to conventional maintenance. Rather than demonstrating the efficacy of TCZ in CPAN, this report proposes a hypothesis-generating, strategy-based therapeutic model that may contribute to optimizing therapeutic exposure by reducing reliance on both GCs and prolonged biologic therapy. Further studies are required to validate this approach.

## Limitations

This report is limited by its descriptive nature as a single-case report, which precludes causal inference. It is not possible to determine whether remission resulted from TCZ, corticosteroids, prior immunosuppressive therapies, or their combination. Although the treatment history makes a delayed effect of MTX or MMF unlikely, this possibility cannot be completely excluded. GCs also represent a potential confounder despite prior lack of sustained response.

In addition, the absence of standardized disease activity measures and limited follow-up restrict conclusions regarding long-term disease control. Therefore, these findings should be interpreted as hypothesis-generating.

## Data Availability

The original contributions presented in the study are included in the article/Supplementary material, further inquiries can be directed to the corresponding author.
